# Tuberculosis screening costs and cost-effectiveness in high-risk groups: a systematic review

**DOI:** 10.1186/s12879-021-06633-3

**Published:** 2021-09-08

**Authors:** H. Alsdurf, B. Empringham, C. Miller, A. Zwerling

**Affiliations:** 1 School of Epidemiology and Public Health, University of Ottawa, 600 Peter Morand Cresent, Ottawa, Canada; 2 Children’s Hospital of Eastern Ontario, Ottawa, Canada; 3 Global TB Programme, World Health Organization, Geneva, Switzerland

**Keywords:** Tuberculosis, Economic evaluation, Systematic review, Cost-effectiveness, Tuberculosis in HIV-infected, Tuberculosis control

## Abstract

**Background:**

Systematic screening for active tuberculosis (TB) is a strategy which requires the health system to seek out individuals, rather than waiting for individuals to self-present with symptoms (i.e., passive case finding). Our review aimed to summarize the current economic evidence and understand the costs and cost-effectiveness of systematic screening approaches among high-risk groups and settings.

**Methods:**

We conducted a systematic review on economic evaluations of screening for TB disease targeting persons with clinical and/or structural risk factors, such as persons living with HIV (PLHIV) or persons experiencing homelessness. We searched three databases for studies published between January 1, 2010 and February 1, 2020. Studies were included if they reported cost and a key outcome measure. Owing to considerable heterogeneity in settings and type of screening strategy, we synthesized data descriptively.

**Results:**

A total of 27 articles were included in our review; 19/27 (70%) took place in high TB burden countries. Seventeen studies took place among persons with clinical risk factors, including 14 among PLHIV, while 13 studies were among persons with structural risk factors. Nine studies reported incremental cost-effectiveness ratios (ICERs) ranging from US$51 to $1980 per disability-adjusted life year (DALY) averted. Screening was most cost-effective among PLHIV. Among persons with clinical and structural risk factors there was limited evidence, but screening was generally not shown to be cost-effective.

**Conclusions:**

Studies showed that screening is most likely to be cost-effective in a high TB prevalence population. Our review highlights that to reach the “missing millions” TB programmes should focus on simple, cheaper initial screening tools (i.e., symptom screen and CXR) followed by molecular diagnostic tools (i.e., Xpert®) among the highest risk groups in the local setting (i.e., PLHIV, urban slums). Programmatic costs greatly impact cost-effectiveness thus future research should provide both fixed and variable costs of screening interventions to improve comparability.

**Supplementary Information:**

The online version contains supplementary material available at 10.1186/s12879-021-06633-3.

## Introduction

The 2020 Global Tuberculosis (TB) Report noted 3 million undiagnosed TB cases annually worldwide [1]. There is an urgent need to strengthen TB programme efforts to improve TB case detection of these missing millions. These efforts involve scaling up systematic screening programmes targeting household contacts and other high-risk groups, including people living with HIV (PLHIV), thereby linking the “missing millions” with diagnosis and treatment. The recent COVID-19 pandemic has dramatically decreased access to TB services and treatment globally, and thus there is an urgent need to get TB programmes back on track to avoid missing even more people who need access to care and treatment.

Passive case finding (PCF) has been the standard for TB diagnosis, and relies on symptomatic individuals self-presenting to healthcare facilities for diagnosis and treatment [2]. Systematic screening is defined by the WHO as the “systematic identification of people at risk for TB disease, in a predetermined target group, by assessing symptoms and using test, examinations or other procedures that can be applied rapidly” [3]. Active case finding (ACF) is a term for systematic screening of people who do not seek out healthcare services [4] and intensive case finding (ICF) is a term for the regular screening for signs and symptoms of TB among people living with HIV [5]. Systematic screening is a broader term which encompasses all TB screening interventions that actively seek to identify people at risk of TB disease. Screening individuals earlier in their disease course decreases the time during which transmission can occur, with the hopes of reducing future TB incidence [6].

Individual programmatic components of systematic screening vary by setting, but may include: door-to-door symptom screening, targeted testing of asymptomatic household contacts of persons with TB disease, or screening campaigns among high-risk subpopulations [7]. The algorithms used for systematic screening can involve different screening tools, such as symptom screening or chest X-ray (CXR), and a variety of diagnostic tools including computer-automated detection (CAD) software for X-ray films, GeneXpert MTB/RIF assay (Xpert^®^), detection of mycobacterial lipoarabinomannan (LAM) antigen in urine, sputum smear microscopy and inflammatory blood work. The success of screening is dependent on both programme and population factors, such as the underlying prevalence of tuberculosis within a population (i.e., pretest probability of detecting TB). The generalizability of one screening strategy to another community cannot be assumed [8].

PLHIV are a key high-risk group for developing TB disease, accounting for approximately 8% of all TB cases globally [1]. Clinical risk factors, like diabetes mellitus, [9] and structural risk factors, such as people residing in prisons, [5] also greatly increase the risk of developing TB and have been suggested as high-risk groups to target for screening programmes. Screening programmes typically incur large costs [10] since high-risk groups for TB are often marginalized or living in difficult to access regions [11].

Economic evaluations may help in ensuring that limited resources are used wisely. The current published literature includes systematic reviews of screening interventions and their economic impact but focused on specific populations (i.e., persons experiencing homelessness or incarceration, PLHIV, immigrants or other high-risk groups) [12–17]. These reviews highlight that although systematic screening and active case finding is recommended in high-risk groups, there is a need for clearer guidance on which specific tools and screening algorithms or strategies are cost-effective, essentially highlighting the gap in knowledge that still exists despite WHO’s endorsement of systematic screening. Two systematic reviews of ICF among PLHIV showed significant variability across countries and target groups of patients, but highlighted that ICF was cost-saving compared to PCF in high TB/HIV burden countries, though authors noted the lack of standardized methods for cost data collection [5, 17]. The other reviews of the cost of TB screening were focused on a specific population and setting (i.e., immigrants in low-TB burden settings or contacts in Eastern Europe) [13–15] and recommended systematic screening for high-risk groups, but noted that there was limited data which was heterogeneous and of low quality. Therefore, our objective was to comprehensively synthesize economic evaluations of systematic screening for TB disease to inform a guideline development meeting leading to the updated guidance on TB screening, “WHO consolidated guidelines on tuberculosis. Module 2: screening—systematic screening for tuberculosis disease” [3] (see full list of PICO questions in the Additional file 1). Our study aimed to provide an up-to-date and comprehensive review of the economic evidence for all systematic screening interventions that was not limited to one sub-population, high-risk group, or setting.

## Methods

### Protocol

We performed a systematic review of the published literature on economic evaluations for TB screening with a focus on high-risk populations of persons with clinical risk factors such as PLHIV, diabetes or other respiratory diseases. We also focused on persons with structural risk factors, defined by the WHO as, “the circumstances in which people are born, grow up, live, work and age,” [18] including persons experiencing homelessness or residing in prisons, miners, elderly or indigenous persons. We sought to understand costs, cost-effectiveness, and affordability of screening approaches in key high-risk populations from the health system perspective. We performed this review according to the PRISMA guidelines (see Additional file 1) [19, 20].

### Information sources

We searched three online databases: Ovid, EMBASE and Scopus for new studies published within the past ten years (i.e., January 1, 2010 through February 1, 2020). This review was intended to inform an update to the current WHO guidelines. Technologies used for screening and diagnosis of TB have significantly improved in recent years, such as the development of rapid molecular tests for TB including Xpert® MTB/RIF (Xpert) and other diagnostics that were not available before 2010. A search strategy was developed to identify cost and cost-effectiveness studies of systematic screening in high-risk groups. We reviewed citations of all eligible articles, guidelines, and reviews for additional studies (see Additional file 1 for search terms).

### Study selection

Studies were included if they evaluated any type of systematic screening activities among persons with clinical or structural risk factors and included costs. Our search terms were designed to broadly capture any economic evaluations or studies including costs and an outcome, such as disability-adjusted life years (DALYs) or quality-adjusted life years (QALYs) and was not limited to cost-effectiveness or cost-utility analyses. Studies including utilities, such as DALYs, without costs were not included. Relevant studies were identified through electronic searches of the online databases, and duplicates were removed. Articles were excluded if they did not evaluate screening activities, or were reviews, letters, or opinion pieces.

### Data collection

Articles were excluded if they only screened for latent TB infection, did not report costs per person screened or diagnosed or did not report the costs for the screening intervention separately from standard care (i.e., PCF). Studies were excluded if they evaluated screening activities in the general population, among contacts or children, since these groups were included in a separate manuscript (accepted, in press). Full text review was done independently by two reviewers (HA and BE) on remaining articles that met predetermined inclusion criteria, with all disagreements resolved by discussion with a third reviewer (AZ). No language filter was applied. Assessment of the quality of each economic evaluation and study quality was guided by the Consensus Health Economic Criteria (CHEC) [21, 22] and CHEERS checklist, respectively (see Additional file 1: Tables S1 and S2) [21].

### Data extraction

The study data elements extracted from each study included: primary research question, country and setting, year of study, patient population, clinical setting, type of intervention, comparison diagnostic scenarios, economic analysis perspective, analytic time horizon, type of economic evaluation, source of costing, primary and secondary outcome measures, type of model, sensitivity and uncertainty analyses performed and willingness-to-pay (WTP) threshold. The WHO’s World Health Report guidelines on Choosing Interventions that are Cost-Effective (CHOICE) are the most commonly referenced WTP threshold among cost-effectiveness studies, particularly in low- and middle-income countries (LMICs) and are typically based on 1–3 times the country gross domestic product (GDP) per capita [23]. However, the use of these GDP-based thresholds has been challenged by many experts, the thresholds are considered overly simplistic and too easily attained when an intervention is effective [24]. Another key criticism of GDP-based WTP thresholds is the lack of their value and usefulness in assessing the trade-offs that decision-makers face in allocating limited healthcare resources [24]. Although the WHO no longer endorses GDP-based WTP thresholds [25], the challenge remains for clinicians, programme managers and researchers to determine the best metric for assessing value and reporting outcomes for cost-effectiveness and affordability of healthcare interventions. For the purposes of this review and to enable comparisons across currently published studies, we have included the GDP-based WTP thresholds used by study authors. However, we support the need for better decision-making tools for resource allocation in the local context, bearing in mind opportunity costs and the burden of disease.

Model parameters were extracted including epidemiologic, treatment and outcome parameters. Key outcomes included: cost per patient diagnosed, and incremental cost-effectiveness ratio (ICER) per utility measure (e.g., DALY averted). Costs are presented in United States Dollars (USD) adjusted to 2019 [27, 28]. Given the heterogeneity of study setting, year and type of screening strategies employed, there was no plan to calculate global estimates or pool data.

## Results

### Study selection

**Fig. 1:**
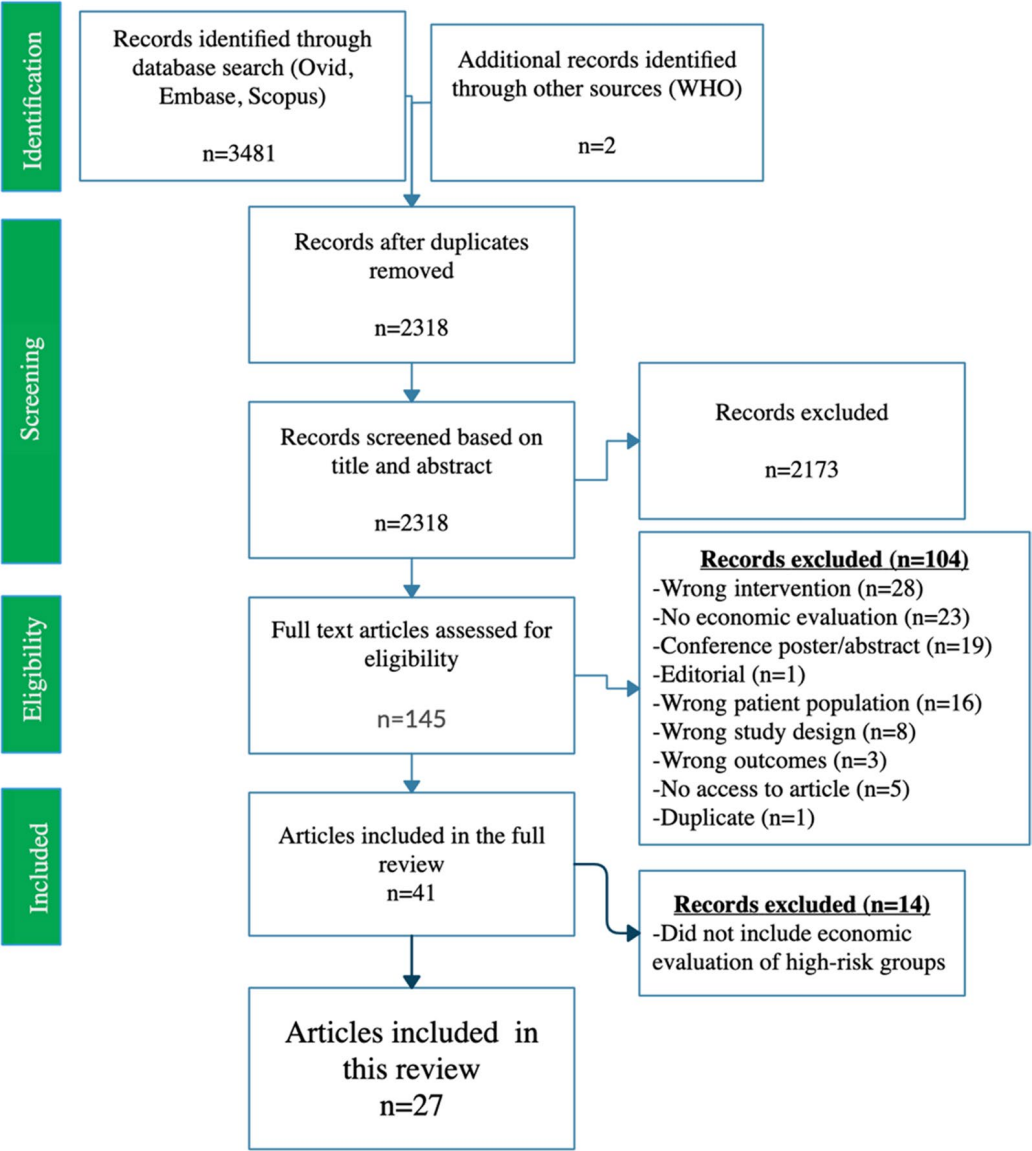
PRISMA diagram

We identified a total of 3481 articles through database searching (Fig. 1). After duplicate removal, we screened 2318 citations by title and abstract for inclusion. Of these, we assessed 145 full-text publications against our inclusion criteria and excluded 118 publications. Exclusions were mainly due to the wrong intervention or no economic evaluation. A total of 27 studies were identified for inclusion in the review [11, 29–53].

### Study characteristics

Study characteristics for the 27 included studies are summarized in Tables 1 and 2; 19/27 (70%) of studies were conducted in high TB burden countries. Some studies included stratified analyses among multiple high-risk populations and thus contributed results to multiple categories (i.e., clinical and/or structural risk factors). Seventeen studies included persons with clinical risk factors; [32, 34, 38] fourteen among PLHIV, the majority of which (12/14) were conducted in Sub-Saharan Africa (SSA) [29–32, 37, 39–42, 44, 45, 49, 52, 53]. Thirteen studies included persons with structural risk factors (i.e., migrants, persons experiencing homelessness, or miners) and were from a range of countries such as Belgium, Cambodia, China, Russia, South Africa, and Zimbabwe [11, 32, 33, 35, 36, 38, 43, 46–48, 51, 54].

**Table 1:** Study Characteristics

Study, year	Country setting	Study population	Screening interventions and diagnostic tools	Reference screening strategies and diagnostic tools	Analysis perspective
Abimbola et al. 2012	South Africa	PLHIV initiating ART	1) WHO 4SS; SSM; SM (−) followed by CXR; CXR (−) followed by culture 2) WHO 4SS; Xpert for diagnosis	Standard practice: SSM; SM (−) followed by CXR	Health system
Adelman et al. 2017	Ethiopia	PLHIV in HIV clinics	WHO 4SS; Xpert for diagnostic test of PLHIV with positive symptom screen (i.e. at least 1 symptom); Xpert( +) then DST/culture	Current recommended practice: 4SS, the SM and/or clinical diagnosis of TB	Health system
Andrews et al. 2012	South Africa	ARV naïve, PLHIV	Initial WHO 4SS then: 1) SSM (2 samples)—Xpert in TB-symptomatic; 2) SSM (2)—Xpert in all; 3) Culture (2)—Xpert in TB-symptomatic; 4) Culture (2)—Xpert in all; 5) Xpert (1) in TB-symptomatic; 6) Xpert (1) in all; 7) Xpert (2) in TB-symptomatic; 8) Xpert (2) in all	No TB screening	Health system
Bassett et al. 2010	South Africa	PLHIV initiating ART	ICF: WHO 4SS and CXR, then all PLHIV provided sputum for QIAmp (PCR) and culture	Symptoms (cough > 2 weeks)	Health system
Bogdanova et al. 2019	Russian Federation	General population, PLHIV, homeless, migrants, chronic medical conditions	1) Contact tracing done then CXR and SSM for diagnosis; 2) Mass screening in hospitals using CXR and SSM; 3) Mass screening in TB dispensary using mobile CXR then SSM	PCF including CXR and SSM	Health system
James et al. 2017	Cambodia	poor, urban residents; elderly living in rural areas	ACF interventions: 1) HOPE—Door-to-door screening WHO 4SS, symptomatic patients SSM (LED), then Xpert and culture; 2) CATA—Door-to-door screening older patients WHO 4SS, if symptomatic referred for CXR screening, if abnormal then Xpert	N/A	Health system
Ji et al. 2020	China	Diabetic patients	ACF intervention—Patients received regular physical exams for 3 years; Diabetes patients all WHO 4SS, then positive symptom had CXR, then SSM, SM(-) then culture for TB confirmation	N/A	Health system
Jit et al. 2011	United Kingdom	Hard to reach individuals	Find and Treat service: 48 mobile CXR screening units offering: 1) mobile CXR; 2) enhanced case management; 3) referral for loss-to-follow-up	PCF – people passively presenting to hospital	Health system
Jo et al. 2020	Cambodia, Tajikistan	Cambodia—elderly, vulnerable groups in rural areas Tajikistan—detention centers and diabetic patients	Cambodia—Community sensitization and training of CHWs, door-to-door WHO 4SS by CHWS, all patients had mobile CXR, abnormal CXR then Xpert Tajikistan—Community sensitization and training of TB staff, CHWs used mobile-phone questionnaire at health facilities WHO 4SS followed by CXR, SSM then Xpert based on SSM results	N/A	Health system
Karki et al. 2017	Papua New Guinea	General population in rural villages	Outreach visits to villages: Systematic 4SS throughout villages, if symptomatic then SSM	N/A	Health system
Kranzer et al. 2012	South Africa	Peri-urban population attending mobile testing	Mobile HIV testing van added TB testing: WHO 4SS for all HIV(-), if symptomatic then SSM; all HIV + SSM and referral to TB clinic for evaluation including CXR	N/A	Health system
Machekera et al. 2019	Zimbabwe	High-risk groups: PLHIV, contacts, miners, HCW, prisoners, elderly	Zimbabwe ACF: WHO 4SS, then all have CXR, if either 4SS or CXR positive then Xpert with clinical diagnosis, if needed	WHO algorithms: WHO2b: 4SS, if positive then Xpert and clinical diagnosis, if needed WHO2d: 4SS, if positive then CXR, then Xpert then clinical diagnosis WHO3b: CXR, then Xpert and clinical diagnosis, if needed	Health system
Maheswaran et al. 2012	Sub-Saharan Africa	PLHIV	1) Any classic symptom; 2) 2 + classic symptoms; 3) CXR on all; 4) SSM on all; 5) 4SS, if positive then CXR; 6) 4SS, if positive then SSM; 7) 4SS, if positive then SSM then CXR; 8) 4SS if positive then CXR then SSM	Chronic cough > 2 weeks	Health system
Murray et al. 2016	Uganda	General population	WHO 4SS, if cough > 2 weeks then triage testing with CXR or CRP then Xpert for diagnosis	PCF (baseline scenario): WHO 4SS, no triage testing, if symptomatic then Xpert	Health system
Orlando et al. 2018	Mozambique	PLHIV	1) Xpert: Xpert for all participants; 2) LAM: urine TB-LAM in all patients with CD4 < 200; Xpert in all patients with CD4 > 200 and TB-LAM(-) with CD4 < 200	Standard: WHO 4SS, if symptomatic then SSM	Health system
Reddy et al. 2019	Malawi, South Africa	Hospitalized PLHIV	ICF intervention: Sputum Xpert, urine LAM and urine Xpert Modified intervention: Sputum Xpert and TB-LAM	Standard of care: Xpert	Health system
Sekandi et al. 2015	Uganda	HHC, urban population	1) PCF + ACF: HCW perform door-to-door chronic cough surveys (> 2 weeks) then collect 2 sputum for SSM, then return test results to the patient at their home; 2) PCF + HHC investigations (HCI): HCW perform WHO 4SS to HHC in home; child contacts and those unable to produce sputum diagnosed using CXR	PCF: Self-referral or presenting to health facility, chronic cough (> 2 weeks). WHO 4SS then SSM, if unable to produce sputum then diagnosed using CXR	Health system, societal perspective
Shah et al. 2017	Peru	HHC (low HIV incidence setting)	1) ACF: PCF + HCW visits to screen HHC using WHO 4SS and SSM; 2) PCF + Xpert; 3) ACF + Xpert: HCW visits to screen HHC using WHO 4SS and Xpert for diagnosis	PCF: Self-referral or presenting to health facility, TB diagnosis using SSM and clinical evaluation	Health system
Shah et al. 2009	Ethiopia	PLHIV (18 + years) in VCT clinic	1) WHO 4SS then SSM, if SM(-) then CXR; 2) CXR for all PLHIV at entry, if CXR( +) then SSM	N/A	Health system
Shah et al. 2008	Vietnam	PLHIV	All PLHIV screened using CXR (all diagnosed HIV + before CXR), confirmed diagnosis with SSM	N/A	Health system
Smit et al. 2017	Belgium	High risk groups and contacts (asylum seekers and migrants)	Systematic screening in high-risk groups including WHO 4SS and CXR; Follow-up of asylum seekers with abnormal CXR, supplemental CXR and periodic screening (6/12 months) after arrival	N/A	Health system
Sohn et al. 2019	India	Rural, tribal population	CHW visits to homes with TB education, screening: WHO 4SS and SSM, CHW return with results	N/A	Health system
Winestsky et al. 2012	Russian Federation	Prisoners	1) CXR screening; 2) WHO 4SS; 3) Annual Xpert screening; 4) WHO 4SS + CXR; 5) CXR + Xpert; 6) WHO 4SS + Xpert; 7) WHO 4SS + CXR + Xpert DST used for treatment planning	PCF: No screening (self-referral)	Health system
Yoon et al. 2019	Uganda	PLHIV in two HIV clinics	Novel ICF algorithms: 1) WHO 4SS + TB-LAM + Xpert; 2) WHO 4SS + TB-LAM + Xpert + culture; 3) POC CRP + Xpert; 4) POC CRP + TB-LAM + Xpert; 5) POC CRP + TB-LAM + Xpert + culture	Current ICF: WHO 4SS, if symptomatic then Xpert for diagnosis	Health system
Zhang et al. 2016	China	High risk groups including elderly (65 +)	1) WHO A1: WHO 4SS, if cough > 2 weeks then CXR, if CXR( +) then SSM; 2) WHO A1B: WHO 4SS, if cough > 2 weeks then CXR, if CXR( +) then SSM; 3) WHO A2: WHO 4SS, if any TB symptoms then CXR, if CXR( +) then SSM; 4) WHO A3: Screening with CXR, if CXR( +) then SSM	N/A	Health system
Zishiri et al. 2014	South Africa	Correctional facility inmates	WHO 4SS questionnaire for all newly admitted inmates, 1 + symptoms then diagnosed with Xpert	N/A	Health system
Zwerling et al. 2015	Malawi	Newly diagnosed PLHIV in rural Malawi	Initial WHO 4SS, if 1 or more TB symptoms, then LED or Xpert; If initial 4SS negative, then patient asked to return 1 month later for second test if still symptomatic; Diagnosis with SSM, LED and Xpert	Standard of care (i.e. discretion of treating physician) using SSM	Health system

*4SS* four symptom screen, *ACF* active case finding, *ART* antiretroviral therapy, *CE* cost effective, *CHW* community health worker, *CRP* C reactive protein, *CXR* chest x-ray, *DALY* disability adjusted life year, *DST* drug-susceptibility testing, *GDP* gross domestic product, *GNI* gross national income, *HCW* healthcare workers, *HHC* household contacts, *HIV* human immunodeficiency virus, *ICER* incremental cost-effectiveness ratio, *ICF* intensified case finding, *PCF* passive case finding, *PLHIV* people living with HIV, *SSM* sputum smear microscopy, *TB* tuberculosis, *USD* United States dollars, *WTP* willingness to pay threshold, *Xpert* GeneXpert MTB/RIF, *YLS* year of life saved

**Table 2:** Study Characteristics of the Economic Evaluation

Study, year	Type of economic evaluation	Empirical study or modelling	Time horizon	Source of costing	Primary outcome	Sensitivity analysis	Key scenarios	WTP threshold
Abimbola et al. 2012	Decision analytic model	Modelling	6 months after ART initiation	South Africa specific published literature	ICER ($/TB death averted)	One-way and probabilistic	Mortality rates, cost inputs	Per-capita South African 2012 GDP ($5,678 USD)
Adelman et al. 2017	Decision analytic model	Modelling	10 years	Parent study results, AHRI HIV clinic, published literature	ICER ($/DALY averted)	One-way	Varied model inputs based on ranges in the literature	Per-capita Ethiopian 2014 GDP ($505 USD)
Andrews et al. 2012	Decision analytic model	Modelling	Lifetime	Cape Town AIDS Cohort, unit costs from hospitals, published literature from South Africa	ICER ($/YLS)	One-way and two-way	Varied key parameters and costs	Per-capita South African 2010 GDP ($7,100 USD)
Bassett et al. 2010	Cost analysis	Empirical	N/A	HIV clinic data (McCord hospital)	Cost ($/TB case identified)	N/A	Subset of patients with cough at study entry	N/A
Bogdanova et al. 2019	Cost analysis	Empirical	N/A	Finance department, medical insurance fund	Cost ($/TB case detected)	N/A	N/A	N/A
James et al. 2017	Cost analysis	Empirical	N/A	CENAT, National TB and Leprosy program, TB REACH	Cost ($/TB case detected)	N/A	N/A	N/A
Ji et al. 2020	Cost-effectiveness analysis; cost-utility and cost benefit analyses	Modelling	3 years	National Essential Public Health Program (NEPHS)	ICER ($/DALY gained)	One-way	Varied key parameters, and ROC curve	3 times per capita 2016 GDP ($ CNY not stated)
Jit et al. 2011	Decision analytic model	Modelling	N/A	Find and Treat program records, NICE guidelines	ICER ($/QALY gained)	One-way	Varied all key parameters	20–30,000 GBP per QALY
Jo et al. 2020	Cost analysis	Empirical	2 years	TB REACH budgets and financial reports, program data	Cost ($/TB case identified)	N/A	N/A	N/A
Karki et al. 2017	Cost analysis	Empirical	N/A	Program expenditures and budget	Cost ($/TB case detected)	N/A	N/A	N/A
Kranzer et al. 2012	Cost analysis	Empirical	20 months	Study data and published literature	Cost ($/TB case detected)	One-way	Varied staff salaries and discount rates	N/A
Machekera et al. 2019	Cost analysis	Empirical	N/A	ZimACF project data	Cost ($/case diagnosed)	One-way	Varied all unit costs	N/A
Maheswaran et al. 2012	Cost-effectiveness analysis	Modelling	2 years	Published data	ICER ($/QALY)	Probabilistic	VOI used as alternative to univariate SA	Per capita 2010 SSA GNI ($2167 USD)
Murray et al. 2016	Decision analytic model	Modelling	Lifetime	Published literature	ICER ($/life year gained)	One-way, multi-way, and probabilistic		Per capita 2014 Ugandan GNI ($680 USD)
Orlando et al. 2018	Cost-effectiveness analysis	Modelling	1 year	DREAM program, Global Fund data, published literature	ICER ($/DALY averted)	One-way	Varied all key parameters	Per capita 2016 Mozambique GDP ($382 USD)
Reddy et al. 2019	Cost-effectiveness analysis	Modelling	2 and 5 years, lifetime	STAMP trial, published literature	ICER ($/Year of life saved (YLS))	One-way, multi-way	Varied all key parameters	Malawi ($750 USD); South Africa ($940 USD)
Sekandi et al. 2015	Cost-effectiveness analysis	Modelling	1.5 years	Uganda NTP, program data and published literature	ICER ($/additional TB case detected)	One-way	Varied costs and probabilities	Twice per capita 2013 Ugandan GDP ($1102 USD)
Shah et al. 2017	Cost-effectiveness analysis	Modelling	N/A	Peru NTP data, published literature	ICER ($/DALY averted)	One-way	Varied all model parameters	Per capita 2014 Peruvian GNI ($6300 USD)
Shah et al. 2009	Cost analysis	Empirical	N/A	Program data	Cost ($/TB case diagnosed)	N/A	N/A	N/A
Shah et al. 2008	Cost analysis	Empirical	N/A	Provincial TB and HIV/AIDS program data	Cost ($/TB case diagnosed)	N/A	N/A	N/A
Smit et al. 2017	Cost-effectiveness analysis	Empirical	1 year	Flemish Association for Respiratory Health and TB Control	ICER (Euros/TB case detected)	One-way	Varied costs and number of cases detected	N/A
Sohn et al. 2019	Cost analysis	Empirical	1 year	Asha Kalp program finance and operations data	Cost ($/TB case detected)	One-way	Top-down and bottom-up cost estimates provided	N/A
Winestsky et al. 2012	Decision analytic model	Modelling	10 years	Primary data in prisons, published literature from Russia, Tajikistan and Latvia	ICER ($/QALY gained)	One-way, selected two-way, situational analyses	Varied all key parameters, and plausible situational analyses	Varied WTP thresholds
Yoon et al. 2019	Cost analysis	Empirical	N/A	Program data	Cost ($/TB case diagnosed)	N/A	N/A	N/A
Zhang et al. 2016	Cost analysis	Empirical	N/A	Program data	Cost ($/TB case diagnosed)	N/A	N/A	N/A
Zishiri et al. 2014	Cost analysis	Empirical	N/A	Department of Correctional Services finance data, published literature	Cost ($/TB case identified)	One-way	Changed base-case parameters	N/A
Zwerling et al. 2015	Decision analytic model	Modelling	Lifetime (assumed 59.2 years)	Cost and operational analysis of four study sites, published literature	ICER ($/DALY averted)	One-way, two-way and probabilistic uncertainty analysis	Varied all key parameters	Per capita 2010 average SSA GDP ($1417 USD)

*4SS* four symptom screen, *ACF* active case finding, *ART* antiretroviral therapy, *CE* cost effective, *CRP* C reactive protein, *CXR* chest x-ray, *DALY* disability adjusted life year, *GDP* gross domestic product, *GNI* gross national income, *HCW* healthcare workers, *HIV* human immunodeficiency virus, *ICER* incremental cost-effectiveness ratio, *ICF* intensified case finding, *PCF* passive case finding, *PLHIV* people living with HIV, *TB* tuberculosis, *USD* United States dollars, *WTP* willingness to pay threshold, *Xpert* GeneXpert MTB/RIF, *YLS* year of life saved

### Study findings

We present the study findings stratified by high-risk subgroup, including persons with clinical and structural risk factors and PLHIV, as well as by screening tool used below.

### Persons with clinical risk factors

Three studies provided cost-effectiveness data for individuals with the following clinical risk factors: diabetes mellitus, chronic respiratory disease and fibrotic lesions (Table 3) [32, 34, 38].

**Table 3:** TB screening algorithm costs among persons with clinical and structural risk factors

First author, Year	Country	Screening algorithm		Source of unit costs	Type of unit costs						Average cost^1^ of screening per person		Average cost^1^ of diagnosis per person	
		Screening	Diagnostic tests		Staff	Equipment	Consumables	Overhead	Transport	TB Treatment				
Persons with structural risk factors (N = 16)^a^														
Migrants, refugees, internally displaced persons (IDPs)														
Bogdanova 2019	Russia	Mass CXR screening	SSM	Reported	✓	✓	✓					$4 per migrant screened		$834 per TB case diagnosed
Smit 2017	Belgium	WHO 4SS, CXR	NR	Calculated		✓	✓					$18 per migrant screened		$506,025 per migrant diagnosed with TB
												NR		$6721 per asylum seeker diagnosed with TB
Homeless persons and intravenous drug users (IDUs)														
Bogdanova 2019	Russia	Mass CXR screening	SSM	Reported	✓	✓	✓					$4-$13		$793 per TB case diagnosed
Jit 2011	United Kingdom	Mobile CXR screening for IDUs and homeless	NR	Reported	✓	✓	✓		✓	✓		NR		$9837 per QALY gained (UR: $6,302-$27,666)
Persons who live in urban slums														
Sekandi 2015	Uganda	WHO 4SS for all HHC	SSM and CXR	Reported	✓	✓	✓		✓			NR		$1371 per additional TB case diagnosed
Shah 2017	Peru	Household visits for WHO 4SS for all HHC	Xpert MTB/RIF	Reported	✓	✓	✓	✓	✓	✓		$32 per person screened		$3244 per DALY averted
James 2017	Cambodia	Door-to-door WHO 4SS	SSM and Xpert	Reported	✓		✓		✓			NR		$268 per TB case diagnosed
Members of tribal or indigenous populations														
Sohn 2019	India	Household visits for TB education and screening	SSM	Reported	✓			✓	✓			< $1 per person screened		$3–5 per TB case diagnosed
Persons residing in prisons														
Machekera 2019	Zimbabwe	WHO 4SS, CXR	Xpert MTB/RIF	Calculated	✓		✓					$14 per person screened		$460 per TB case diagnosed
Smit 2017	Belgium	WHO 4SS, CXR	NR	Calculated		✓	✓					$18 per person screened		$14,034 per TB case diagnosed
Winetsky 2012	Former Soviet Union	WHO 4SS alone	NR	Reported	✓	✓	✓	✓				$3 per person screened		$538 per QALY gained
		MMR (CXR)	NR	Reported	✓	✓	✓	✓				$5 per person screened		
		Xpert MTB/RIF	NR	Reported	✓	✓	✓	✓				$2 per person screened		
Zishiri 2014	South Africa	WHO 4SS	Xpert MTB/RIF	Reported	✓	✓	✓	✓		✓		$33 per person screened		$1423 TB case diagnosed
Elderly (55 +)														
Jo 2020	Cambodia (> 55)	Symptom screen	Xpert, SSM, CXR	Reported	✓	✓	✓	✓	✓			< $1 per person screened		$406 per TB case diagnosed
James 2017	Cambodia (> 55)	Symptom screen	Xpert, CXR	Calculated	✓	✓	✓	✓				< $2 per person screened		$340 per TB case diagnosed
Zhang 2017	China (> 65)	Door-to-door symptom screen or CXR	CXR, SSM, culture	Reported	✓	✓	✓	✓				NR		$74–$315 per TB case diagnosed
People living in areas with limited access to healthcare (remote, isolated, hard-to-reach areas)														
Karki 2017	Papua New Guinea	Community-wide symptom screening	SSM	Reported	✓		✓		✓			NR		$158 per TB case diagnosed
Jo 2020	Cambodia	Symptom screen	Xpert, SSM, CXR	Reported	✓	✓	✓	✓	✓			< $1 per person screened		$406 per TB case diagnosed
Miners (i.e., workers with silica exposure)														
Machekera 2019	Zimbabwe	WHO 4SS, CXR	Xpert MTB/RIF	Calculated	✓		✓					$14 per person screened		$404 per miner diagnosed
Persons with clinical risk factors (N = 3)^b^														
Diabetes mellitus														
Bogdonova 2019	Russia	CXR	SSM	Reported	✓		✓		✓			NR		$21,780 per TB case diagnosed
Ji 2020	China	CXR	SSM	Calculated	✓	✓	✓		✓			< $2 per person screened		$288 per DALY averted
Machekera 2019	Zimbabwe	CXR	Xpert	Reported	✓	✓	✓					NR		$2191 per TB case diagnosed
Respiratory disease														
Bogdonova 2019	Russia	CXR	SSM	Reported	✓		✓		✓			NR		$32,746 per TB case diagnosed
Gastro-intestinal, genito-urinary, steroid use or fibrotic chest lesions														
Bogdonova 2019	Russia	CXR	SSM	Reported	✓		✓		✓			NR		$11,648-$105,754 per TB case diagnosed

^a^There is limited evidence on screening among persons with structural risk factors, including migrants (n = 2), homeless persons and IDUs (n = 2), persons who live in urban slums (n = 3), members of tribal or indigenous populations (n = 1), persons residing in prisons (4), elderly (3), people living in remote areas (n = 2) and miners (1). The costs of screening among persons with structural risk factors ranged from $1–33 per person screened and $3–$506,025 per TB case diagnosed. Screening programs were found to be cost-effective in persons living in urban slums and homeless, with reported ICERs of $3244 per DALY averted and $9837 per QALY, respectively. Screening was not shown to be cost-effective in migrants with an of ICER $506,025 per migrant diagnosed. In the Former Soviet Union, screening persons residing in prisons was found to be cost-effective with an ICER of $538 per QALY gained. Door-to-door screening of the elderly in China was shown to be cost-effective with ICERs ranging from US$74 to $315 per additional TB patient diagnosed

^b^There is limited evidence on screening among persons with clinical risk factors, primarily from one study in Russia. One study in Russia demonstrated a cost ranging from $11,648 to $105,754 for systematic screening among persons with various clinical risk factors (i.e., gastro-intestinal, respiratory disease, genito-urinary, steroid use and fibrotic chest lesions). Two studies conducted in Russia and Zimbabwe reported the cost per person diagnosed with diabetes mellitus (DM) which ranged from $2191 to $21,780. A cost-effectiveness analysis of patients with DM in China found systematic screening using CXR was cost-effective with an ICER of $288 per DALY averted

*ACF* active case finding, *HHC* household contacts, *SSM* sputum smear microscopy, *Xpert* GeneXpert, *WHO 4SS* four symptom screen, *mWRDs* molecular WHO-approved rapid diagnostics, *CXR* chest x-ray, *NR* not reported

^1^Costs in 2019 USD unless stated otherwise

**‘**✓**’** indicates cost component was explicitly included in unit cost calculation

### Diabetes and other medical co-morbidities

Bogdanova et al. assessed screening with chest X-ray (CXR) in Russia, and stratified results among diabetic patients as well as persons with other medical comorbidities (i.e., respiratory disease, gastro-intestinal, or fibrotic chest lesions); [32] reported costs ranged from US$11,648 to $105,754 per TB case diagnosed [32]. In Zimbabwe, Machekera et al. found that screening diabetic patients with CXR followed by Xpert® cost US$2191 per person diagnosed with TB [38]. Ji et al. reported that routine screening with CXR among Chinese patients with diabetes was considered highly cost-effective, as compared to PCF, with an ICER of US$288 per DALY averted [34]. Machekera et al. included personnel and laboratory costs and had a large number needed to screen which drove up the cost per person diagnosed, while Ji et al. did not include the overhead costs for diabetic patient care.

### People living with HIV (PLHIV)

There was significant heterogeneity of screening and diagnostic tools used among the 14 studies reporting the cost and cost-effectiveness of programmes in PLHIV (Table 4). Studies often considered multiple diagnostic algorithms or tools. Seven studies used molecular rapid diagnostic tests (i.e., Xpert®MTB/RIF) as an initial screening tool; [29–31, 40–42, 49, 52, 53] six studies used CXR to screen PLHIV in the outpatient setting; [31, 32, 39, 40, 44, 45] two studies used C-reactive protein (CRP) to screen; [40, 49] and one study used sputum smear microscopy (SSM) alone for screening [37].

**Table 4:** TB Screening Algorithm Costs in PLHIV

First author, Year	Country	Screening algorithm		Source of Unit Costs	Type of unit costs					Average cost^1^^,2^ of the screening algorithm per person	Average cost^1^^,2^ of diagnosis per person
		Screening	Diagnostic Tests		Staff	Equipment	Consumables	Overhead	Transport		
PLHIV—using molecular rapid diagnostic tests (i.e., Xpert MTB/RIF) for screening (N = 7)^a^											
Bassett 2010	South Africa	WHO 4SS, sputum	Xpert MTB/RIF	Reported by study	✓	✓	✓			$52 per person screened	$324 per additional TB case diagnosed
Andrews 2012	South Africa	Two Xperts for everyone	Xpert MTB/RIF	Reported by study	✓	✓	✓			$37 per person screened	$4,096 per YLS
Abimbola 2012	South Africa	WHO 4SS	Xpert MTB/RIF	Reported by study		✓	✓			$26 per person screened	$48,542 per death averted
Zwerling 2015	Malawi	WHO 4SS, Xpert	Xpert MTB/RIF	Reported by study	✓	✓	✓	✓		50 tests/year: $116 per person screened	$1,980 per DALY averted (UR: $1544-$3552)
										1000 tests/year: $18 per person screened	$398 per DALY averted (UR: $80–1682)
Adelman 2017	Ethiopia	WHO 4SS, Xpert	Xpert MTB/RIF	Reported by study	✓	✓	✓			NR	$5 per death averted
Orlando 2018	Mozambique	Screening with Xpert	Xpert MTB/RIF	Reported by study		✓	✓			$10 per person screened	$37 per DALY averted
		CD4 count < 200 then LF-LAM	Xpert; LF-LAM	Reported by study		✓	✓			$3 per person screened	$51 per DALY averted
Reddy 2019	South Africa	Sputum/urine Xpert, TB-LAM	Xpert MTB/RIF	Calculated from study data		✓	✓			$31 per person screened* (Range: $11-$73)	$802 per YLS
	Malawi									$72 per person screened* (Range: $18-$105)	$596 per YLS
PLHIV—using CXR for screening (N = 6)^b^											
Shah 2008	Vietnam	Monthly home visits to PLHIV with a CXR voucher	NR	Calculated from study data	✓	✓	✓		✓	$7-$11 per person screened	$115 per TB case diagnosed
Shah 2009	Ethiopia	CXR for all PLHIV at entry to the clinic	SSM on all positive CXR	Calculated from study data		✓	✓			$6 per person screened	$106 per TB case diagnosed
Bassett 2010	South Africa	WHO 4SS, CXR	Xpert MTB/RIF	Reported by study	✓	✓	✓			$52 per person screened	$324 per additional TB case diagnosed
Mahesawaran 2012	Sub-Saharan Africa (SSA)	Symptom screen for any symptom	SSM then CXR if smear negative	Reported by study		✓	✓			$11 per person screened (Range: $10-$29)	$6,245 per QALY (UR: $6,245-$19,581)
Murray 2016	Uganda	Screening for cough, CXR triage test	Xpert MTB/RIF	Reported by study		✓	✓			$22 per person screened	$536 per YLG (UR: $176-$2,514)
Bogdanova 2019	Russian Federation	Mass CXR screening programs in PLHIV	NR	Calculated from study data	✓	✓	✓		✓	$4-$7 per person screened	$570 per TB case diagnosed
PLHIV—using CRP for screening (N = 2)^c^											
Murray 2016	Uganda	Screening for cough, CRP triage test	Xpert MTB/RIF	Reported by study	✓	✓				$21 per person screened	$517 per YLG (UR: $194-$1,535)
Yoon 2019	Uganda	POC CRP; TB-LAM	Xpert MTB/RIF	Calculated from study data		✓	✓			$13-$34 per person screened	$69-$92 per additional TB case diagnosed
Outpatient PLHIV—SSM only for screening (N = 1)^d^											
Kranzer 2012	South Africa	WHO 4SS; SSM at a mobile HIV testing clinic	SSM	Reported by study	✓	✓	✓			NR	$762 per TB case diagnosed

^**a**^In Sub-Saharan Africa, the average cost per person screened using rapid molecular diagnostic tests ranged from $3 to $116. Screening programs were found to be cost-effective in 6/7 (86%) of studies, with the following ICERs reported: $324 per additional TB case diagnosed; $37-$79 per DALY averted; $596–$4096 per YLS; and $517 per YLG. However, one study in Malawi found that screening with Xpert was not cost-effective at low test volumes, with an ICER of $1,980 per DALY averted. Cost-effectiveness depended on the volume of tests conducted annually and prevalence of TB

^**b**^Data was available for among outpatient PLHIV using CXR as a screening tool from six studies of good quality overall. Three studies, from South Africa, Russia and Vietnam, included health system costs as well as unit tests costs, and reported an average cost per person screened using CXR ranging from $4 to $29. Three studies in Sub-Saharan Africa reported the average cost per person screened using CXR ranged from $6 to $52. The average cost per TB case diagnosed ranged from $106-$570. Screening with CXR was shown to be cost-effective among PLHIV with ICERs of $6245 per QALY and $536 per YLS

^**c**^Limited data was available for screening among outpatient PLHIV using CRPs from two studies both of which were of good quality. The average cost for test costs alone of outpatient PLHIV in Uganda screened using CRP with Xpert, TB-LAM and culture for diagnosis ranged from $13 to $34 per person screened and was shown to be cost-effective with an ICER of $517 per YLS or $69–$92 per additional TB case diagnosed in Uganda

^**d**^Limited data was available from one study conducted at an HIV testing clinic in South Africa for screening using SSM alone. This study reported an average cost of $762 per TB case diagnosed at a mobile testing clinic

*CRP* C-reactive protein, *CXR* chest x-ray, *mWRDs* molecular WHO-approved rapid diagnostics, *NR* not reported, *PLHIV* people living with HIV, *POC* point-of-care, *WHO 4SS*, four symptom screen, *Xpert* GeneXpert, *YLG* year of life gained, *YLS* year of life saved

^1^Costs in 2019 USD unless stated otherwise

^2^All costs reported in 2019 USD unless stated otherwise

### Screening PLHIV with molecular rapid diagnostics

All seven studies that assessed screening in PLHIV using Xpert® MTB/RIF as an initial test were conducted in Sub-Saharan Africa, with 6/7 concluding screening was cost-effective. Two studies among PLHIV in South Africa conducted initial WHO-recommended four symptom screen (W4SS), including screening for cough of any duration, weight loss, fever or night sweats [55], followed by screening with Xpert® and found screening to be cost-effective with ICERs of US$324 per additional TB patient diagnosed and US$48,542 per TB death averted [29, 31]. Andrews et al. used two Xperts® to screen all PLHIV at a clinic in South Africa which was found to be cost-effective with an ICER of $4,096 per year of life saved (YLS) (WTP threshold: $7100 per YLS). [30] Among PLHIV initiating anti-retroviral therapy (ART), a combination of HIV treatment medications, in Mozambique, Orlando et al. showed that W4SS followed by screening with either Xpert® alone or Xpert® and lateral flow urine lipoarabinomannan assay (LF-LAM) was cost-effective, compared to W4SS and SSM, with ICERs ranging from US$37 to $51 per DALY averted [41]. Reddy et al. modeled the impact of a screening intervention with TB-LAM, urine and sputum Xpert® in hospitalized PLHIV, regardless of symptoms, in Malawi and South Africa [42]. The intervention was cost-effective, compared to sputum Xpert® alone (standard of care), with reported ICERs of US$450 and US$840 per YLS in Malawi and South Africa (WTP threshold: $750 and $940 per YLS, respectively).

Zwerling et al. found a randomized controlled trial using point of care (POC) Xpert® to screen PLHIV in rural Malawi was not cost-effective, due to low-test volumes (i.e., 50 tests/year, ICER of US$1980 per DALY averted, Uncertainty Range (UR): $1544–$3552). Zwerling et al. showed that Xpert® could be cost-effective at higher-test volumes (1000 tests/year, ICERs of US$398 per DALY averted, UR: $80–$1682) [WTP threshold: 3 × GDP per capita of Malawi (US$254)].

### Screening PLHIV with CXR

Four of the six studies (67%) reporting on the use of CXR to screen PLHIV were conducted in SSA. The cost per person diagnosed with TB ranged from US$106 to $570 [31, 32, 44, 45]. Murray et al. found that community screening for cough followed by CXR in Uganda was cost-effective with an ICER of US$536 per year of life gained (YLG) (UR: $176-$2514) [40] Mahesawaran et al. showed the W4SS followed by CXR in SSA was cost-effective with an ICER of US$6245 per QALY (UR: $6245–$19,581) [WTP threshold: 3 × GNI per capita (US$2167)] [39].

### Screening PLHIV with CRP

Among ART-naïve HIV clinic patients in Uganda, Yoon et al. found POC CRP followed by Xpert® for diagnosis [49] algorithms were cost-saving compared to W4SS followed by Xpert® (standard of care), ranging from US$69 to $92 per TB patient diagnosed. Murray et al. showed that CRP was cost-effective as a triage test in PLHIV in Uganda, compared to Xpert®, with an ICER of $517 per YLG (UR: $176–$2514) [WTP threshold: Ugandan per capita GDP (US$609)]. [40].

### Screening among PLHIV using SSM

Kranzer et al. performed an intervention of adding TB symptom screening and SSM to an existing mobile HIV testing clinic in South Africa, [37] with an average cost of US$762 per TB case diagnosed [37].

### Persons with structural risk factors

Thirteen studies provided cost effectiveness data for individuals with structural risk factors including (Table 3): migrants, [32, 47] persons experiencing homeless, [11, 32] persons who live in urban slums, [33, 43, 46] members of indigenous populations, [54] persons residing in prison, [38, 47, 48, 51] elderly, [33, 35, 50] people living in remote areas, [35, 36] and miners [38].

### Migrants, persons experiencing homelessness and intravenous drug users (IDUs)

Bogdanova et al. assessed the cost of screening with CXR in Russia in multiple subgroups and found a cost of US$834 and US$793 per migrant and homeless person diagnosed with TB, respectively [32]. Jit et al. demonstrated that a programme using mobile screening vans among persons experiencing homelessness and IDUs in London, United Kingdom was cost-effective, compared to PCF, with an ICER of USD $9837 (UR: $6301–$28,666) per QALY gained (WTP threshold: £20,000–£30,000 per QALY gained set by NICE standards) [11]. However, in Belgium, Smit et al. showed CXR screening among migrants from high TB incidence countries was not cost-effective, compared with PCF, with an ICER of $506,025 (95% UR: $90,686-$2,040,006) per additional TB case diagnosed [47].

### Persons who live in urban slums

Three studies examined door-to-door screening interventions in urban slums. James et al. reported that door-to-door symptom screening in Cambodian slums using CXR followed by Xpert® for diagnosis cost US$268 per TB case diagnosed [33]. Shah et al. demonstrated that household visits to screen all contacts of persons with TB in an urban slum in Lima, Peru was cost-effective, compared to PCF, with an ICER of US$3244 per DALY averted [WTP threshold: 2014 per capita GNI for Peru (US$6360)]. [46] However, Sekandi et al. found that door-to-door symptom screening of all household contacts, followed by SSM and CXR in urban Uganda, was not cost-effective compared to PCF with an ICER of US$1371 per additional TB case diagnosed [WTP threshold: twice the 2012 Ugandan per capita GDP (US$551)]. [43].

### Members of indigenous populations

Sohn et al. reported a cost of US$3–$5 per person detected with TB for a programme of home visits by community health workers (CHWs) to indigenous persons in rural India. This study included the costs for CHWs time to travel to homes, conduct screening and diagnostic visits, provide directly observed therapy (DOT) and care, as well as the laboratory and administrative services, [54] but did not account for routine TB care and medications provided by the Indian government.

### Persons residing in prisons

Four studies provided direct evidence for the costs of screening among persons residing in prisons [38, 47, 48, 51]. Zishiri et al. found W4SS followed by Xpert® for all persons residing in prison in South Africa cost US$1423 per person diagnosed. [51] While in Zimbabwe, Machekera et al. reported that an intervention of W4SS and CXR followed by Xpert® cost US$460 per person diagnosed [38]. Winetsky et al. performed a dynamic transmission model of TB among persons residing in prisons in the Former Soviet Union and demonstrated that annual screening with Xpert® was cost-effective compared with mass CXR screening, with an ICER of US$538 per QALY gained [WTP threshold: per capita GDP of Tajikistan (US$1900)]. [48] Smit et al. demonstrated that systematic screening of persons residing in prisons using W4SS followed by CXR in Belgium was cost-effective, compared to PCF, with an ICER of US$14,034 (95% UR: $10,898–$18,033) per additional case of TB detected. [47].

### Elderly

Two studies were conducted in Cambodia of door-to-door screening using the W4SS followed by CXR and Xpert® for diagnosis among the elderly (55 +), and reported average costs ranging from US$340 to $406 per person diagnosed with TB [33, 35]. Zhang et al. found that door-to-door symptom screening and CXR among the elderly in China (65 +) had costs ranging from US$74-$315 per TB case diagnosed [50]. Among elderly persons in China, additional risk factors (i.e., male, tobacco use or close TB contact) were associated with higher average costs per patient diagnosed [50].

### People living in remote areas

In Papua New Guinea, Karki et al. reported that an intervention using SSM to screen all villagers with symptoms of TB cost US$158 per person detected with TB. [36] A second intervention, conducted by Jo et al. in remote Cambodia, included house-to-house symptom screening followed by mobile CXR and reported a cost of US$406 per person diagnosed with TB [35].

### Miners

There was limited evidence for screening in miners from one study in Zimbabwe. [38] Machekera et al. demonstrated that a screening algorithm of CXR followed by Xpert® among those with positive CXR was cost saving, with a cost of US$404 compared to US$576 per person diagnosed with an algorithm screening everyone with W4SS and CXR.

## Discussion

Our review of the published literature identified 27 studies of systematic screening among high-risk groups for TB, such as PLHIV, miners, persons residing in prisons, and the elderly. Our review found that systematic screening approaches are most likely to be cost-effective in settings and or populations with high prevalence of TB, such as PLHIV, persons residing in prisons and urban slum dwellers. Studies demonstrated that initial screening with more costly diagnostic tests was not cost-effective in high-risk groups, except among PLHIV. Simple, inexpensive initial screening methods (i.e., W4SS or CXR) followed by molecular diagnostic tools (i.e., Xpert®) among the highest risk groups in the local setting are the most cost-effective approaches to systematic screening. In high TB prevalence settings, door-to-door symptom screening in densely populated areas (i.e., slums), was generally shown to be cost-effective. However, mobile CXR units were not cost-effective due to high programmatic costs, particularly when interventions were targeting hard to reach populations (i.e., persons experiencing homelessness or IDUs). There was limited evidence identified for each high-risk group included in this review, thus caution should be used when extrapolating from a small number of studies.

Our review found the most evidence for cost-effectiveness of screening programmes among PLHIV. Despite varying screening strategies (i.e., W4SS, CRP, CXR, Xpert® MTB/RIF) and patient settings (i.e., in-patient or outpatient clinic), the majority 9/10 (90%) of PLHIV studies that calculated an ICER found screening interventions to be cost-effective among PLHIV using an author determined WTP threshold. Key drivers of costs among PLHIV included: annual test volume and diagnostic test costs, [52] underlying prevalence of TB and HIV, [42, 52] clinic setting, [32, 44, 45] and programmatic costs included (i.e., transportation, mobile van units, or staffing costs) [31, 32, 55]. In sensitivity analyses of low-TB prevalence settings among PLHIV, screening strategies with simple tools, such as W4SS and SSM, were cost-saving compared to more expensive tools such as CXR or Xpert®, [39] highlighting the importance of the local setting.

Screening interventions in high TB prevalence settings, such as urban slums or among persons residing in prisons, increased identification and diagnosis of people with TB and were shown to be cost-effective [46, 48]. Equally important was ensuring proper follow-up to avoid treatment failure or loss post-screening [44]. However, among groups with other medical conditions, high programmatic screening costs, coupled with low TB prevalence meant screening interventions were not cost-effective, but was limited to two studies [32, 38].

Systematic screening is an expensive undertaking, particularly compared to PCF, when it involves mobilizing staff to go into the community. Evidence from this review suggests that community-based interventions [43] had higher costs compared to systematic screening programmes targeting persons presenting to healthcare facilities [35]. The use of artificial intelligence (AI) to inform screening tools through the use of CAD software is an exciting prospect for improving the efficiency and affordability of screening from standard CXR. However, our review did not find any studies which used CAD software in the context of a systematic TB screening programme. Indirect evidence, not presented in this review, has shown that the unit costs for each CXR read with CAD software are likely to be small but require significant investment in equipment and maintenance costs as well as the purchase of CAD software. These new technologies are still being evaluated in many programmes and highlight the need for costing and cost-effectiveness studies to inform their use in the programmatic setting.

The WTP thresholds, which are noted a priori by study authors and based on WHO recommendations, determine whether a given ICER is considered cost-effective. Among the papers reviewed in this analysis, many used either a country’s GDP, or twice the GDP, as the WTP threshold, however there was significant heterogeneity due to the range of country settings. This variability makes comparisons challenging, particularly since setting a higher threshold increases the likelihood of an intervention being considered cost-effective. Careful attention should be paid to the WTP threshold employed by study authors when interpreting cost-effectiveness. Furthermore, there are concerns about using GDP per capita as the basis of determining cost-effectiveness, particularly in LMICs where there are more stringent resources constraints [23]. A key concern is that using a threshold that is too low (i.e., GDP per capita) may result in health systems choosing not to adopt an intervention that would generate net health benefits because the threshold does not take into account health opportunity costs [56]. Current efforts to develop country-specific estimates that account for opportunity costs, as well as updated data on population and economic growth, are underway and aim to provide better options for informing decision-making and resource allocation for health interventions [24, 25, 56].

### Limitations

The heterogeneity around reporting of costs and costing components made comparisons across studies challenging. For instance, some costing analyses accounted for all operational and personnel-related costs, and thus reported higher total costs, while other analyses report only direct costs related to diagnostics testing [11, 38]. There were no standardized screening algorithms, even across similar high-risk groups, and employed different standards of care (i.e., PCF or symptom screen alone or with CXR) which limits comparability of studies and generalizability to other settings. Not all studies describe cost components in the same manner [11] and comparisons across studies is further impeded by a range of primary outcomes from cost per case diagnosed, $/DALYs averted, $/QALY, or $/YLS. Thus, even among the studies that do calculate an ICER, direct comparison is not necessarily appropriate. No included studies assessed the impacts of screening on earlier case detection and proper TB treatment, but this is an area that merits additional evidence to better understand the impact on cost and cost-effectiveness of preventing additional disease transmission.

Our study was restricted to the published literature and thus is likely impacted by publication bias towards those interventions that were shown to be cost-effective; programmes that were not deemed cost-effective may not have been published. A recent Task Force Report from the Professional Society for Health Economics and Outcomes Research (ISPOR) suggests benchmarking approaches, such as reviewing trial protocols, to better explore the potential for publication bias but also notes the need to develop new approaches to assess publication bias [57]. Further, more recent economic evaluations of screening interventions, particularly for novel diagnostic tests such as Xpert®, may have been more likely to demonstrate cost effectiveness than earlier studies due to consistently decreasing test costs.

Our review highlights key gaps in the existing economic evidence, namely the need for more studies on the costs and cost-effectiveness of systematic TB screening programmes from Latin America and Asia, since the majority of included studies took place in SSA. Access to various screening and diagnostic tools was not consistent across study settings. Increased efforts should be made to ensure availability of newer diagnostic technologies to TB programmes globally. In addition, standardization of systematic screening interventions, along with fixed and variable costs included in economic analyses of programmes, is needed for better evidence generation and comparability across studies.

## Conclusions

The COVID-19 pandemic has dramatically impacted TB services globally. Modelling has shown that COVID-19 related restrictions and interruptions to TB programmes may result in an increase in TB incidence up to 6.3 million, and mortality to 1.5 million, by 2025 [58, 59]. Our review is the first to summarize the economic evidence for systematic screening for TB disease among high-risk groups. Our review highlights that to reach the “missing millions”, and address the setbacks to TB services due to the COVID-19 pandemic, TB programmes should focus on simple, cheaper initial screening tools (i.e., symptom screen and CXR) followed by molecular diagnostic tools (i.e., Xpert®) among the highest risk groups in the local setting (i.e., PLHIV, urban slums). Programmatic costs greatly impact cost-effectiveness thus future research should provide both fixed and variable costs of screening interventions to improve comparability. COVID-19 has dramatically increased the number of digital applications for contact screening, as well as other video or telehealth options for service delivery. Expanded use of such digital technologies can be leveraged for TB screening to improve identification and treatment options for patients globally.

## Supplementary Information

**Additional file 1.Annex S1.** Key PICO questions and full search terms used in systematic review.

## Data Availability

Data sharing is not applicable to this article as no datasets were generated or analyzed during the current study.

## Abbreviations

W4SS: WHO-recommended four symptom screen
CRP: C-reactive protein
CXR: Chest x-ray
DALY: Disability-adjusted life year
DM: Diabetes mellitus
DOT: Directly-observed therapy
GDP: Gross domestic product
GNI: Gross national income
ICER: Incremental cost-effectiveness ratio
PCF: Passive case finding
PLHIV: People living with HIV
POC: Point of care
QALY: Quality-adjusted life year
SSA: Sub-Saharan Africa
SSM: Sputum smear microscopy
TB: Tuberculosis
WHO: World Health Organization
UR: Uncertainty range
USD: United States dollar
WTP: Willingness-to-pay
Xpert^®^: GeneXpert^®^
YLG: Year of life gained
YLS: Year of life saved
