# Supplementary material for: Tuberculosis screening costs and cost-effectiveness in high-risk groups: a systematic review
Source: BMC Infect Dis. 2021 Sep 8;21:935. doi: 10.1186/s12879-021-06633-3 (PMC8425319; doi:10.1186/s12879-021-06633-3)
Supplement: Supplementary file 1 — Additional file 1.Annex S1. Key PICO questions and full search terms used in systematic review. [file 12879_2021_6633_MOESM1_ESM.docx]

**Tuberculosis screening costs and cost-effectiveness in high-risk groups: A systematic review**

**H. Alsdurf^1^, B. Empringham^1^, A. Zwerling^1^,**

**Corresponding author: A. Zwerling,** [**azwerlin@uottawa.ca**](mailto:azwerlin@uottawa.ca)**, 600 Peter Morand Cresent, Ottawa, Canada**

**Affiliations**

1. University of Ottawa, School of Epidemiology and Public Health, Ottawa, Canada

**Disclaimer:** The manuscript is an original submission. The views expressed represent the views of the authors and are not an official position of the above institutions.

**Sources of support: Canadian Institutes of Health Research**

**Abstract**: 295

**Word count:** 3,015

**Number of Tables:** 4 tables

**Number of Figures:** 1 figure

**Keywords:** Tuberculosis, economic evaluation, systematic review, cost-effectiveness, tuberculosis in HIV-infected, tuberculosis control

Supplemental File

**Key PICO questions being addressed are as follows:**

1. Should molecular rapid diagnostics be used to screen for pulmonary TB in miners against a microbiological reference standard?
2. Should molecular rapid diagnostics be used to screen for pulmonary TB in PLHIV, irrespective of signs and symptoms of TB, against a microbiological reference standard?
3. Should a C-Reactive Protein (CRP) cutoff of 10 mg/L vs. WHO-recommended four symptom screen (WHO 4SS) be used to screen for active TB in outpatient PLHIV (not on ART)?
4. Should molecular rapid diagnostics vs. WHO 4SS be used to screen for active TB in outpatient PLHIV (not on ART)?
5. Should CXR (any abnormality) vs. WHO 4SS be used to screen for active TB in outpatient PLHIV (not on ART)?
6. Should molecular rapid diagnostics vs. WHO 4SS be used to screen for active TB in inpatient PLHIV (regardless of ARV)?
7. Should molecular rapid diagnostics vs. WHO 4SS be used to screen for active TB in pregnant women with HIV (regardless of ARV)?
8. Should systematic screening for TB be conducted for workers with silica exposure (miners)?
9. Should systematic screening for TB be conducted for prisoners versus standard case detection?
10. Should systematic screening for TB be conducted for persons with clinical risk factors versus standard case detection (*among persons who are HIV-negative or unknown HIV status*)?
    - Persons with diabetes mellitus
    - Persons with substance use disorder
    - Persons with fibrotic lesions on chest x-ray
    - Persons previously treated for TB
    - Persons with chronic respiratory disease
    - Smokers
    - Persons who are undernourished
    - People with immunocompromising conditions (solid organ transplant recipients, chronic renal failure or hemodialysis)
    - Pregnant persons
11. Should systematic screening for TB be conducted for persons with structural risk factors versus standard case detection?
    - Migrants, refugees, internally displaced persons (IDPs)
    - Homeless persons
    - Persons who live in urban slums
    - Members of tribal or indigenous populations
    - People living in areas with limited access to health care (remote, isolated, hard-to-reach areas)

**Search Terms:**

The search included the terms: “tuberculosis”, “latent tuberculosis”, “extensively drug-resistant tuberculosis”, “*Mycobacterium tuberculosis*”, “tuberculosis, pulmonary” AND “active case finding”, “intensified case finding”, “tuberculosis case finding”, “mass screening”, “mass chest x-ray”, “multiphasic screening”, “triage OR screening” AND “cost-benefit”, “cost”, “economic”, “cost effectiveness”, “cost-utility”, “disability adjusted life year”, “DALY”, “quality-adjusted life year”, “QALY”, “cost benefit analysis”, “cost effectiveness analysis”, “quality of life”, or “utility”. Search terms were adapted slightly for each database as required.

Search Strategy:

(tb or tuberculosis).mp OR Latent Tuberculosis/ or Extensively Drug-Resistant Tuberculosis/ or Tuberculosis/ or Mycobacterium tuberculosis/ or Tuberculosis, Pulmonary/ or Mycobacterium/ or Tb/

AND ((active adj3 case finding) or intens*adj 3 case finding).mp. OR (tuberculosis adj3 case finding).mp. OR mass screening/ or mass chest x-ray/ or multiphasic screening/ OR triage.mp OR screening.mp

AND (cost-benefit or cost or economic or cost effectiveness or cost-utility or disability adjusted life year or DALY or quality-adjusted life year or QALY or cost benefit analysis or cost effectiveness analysis or quality of life or utility).mp

Supplemental Table 1: Quality Assessment Part I

| **Study Author** | **Is the study population clearly described?** | **Are competing alternatives clearly described?** | **Is a well-defined research question posed in answerable form?** | **Is the economic study design appropriate to the stated objective?** | **Is the chosen time horizon appropriate in order to include relevant costs and consequences?** | **Is the actual perspective chosen appropriate?** | **Are all important and relevant costs for each alternative identified?** | **Are all costs measured appropriately in physical units?** | **Are costs valued appropriately?** |
| --- | --- | --- | --- | --- | --- | --- | --- | --- | --- |
| Abimbola et. al | Yes | Yes | Yes | Yes | No | Yes | Yes | Yes | Yes |
| Adelman et al. | Yes | Yes | Yes | Yes | Yes | Yes | Yes | Yes | Yes |
| Andrews et. al | Yes | Yes | Yes | Yes | Yes | Yes | Yes | Yes | Yes |
| Bassett et. al | Yes | Yes | Yes | Yes | No | Yes | Yes | Yes | Yes |
| Bogdanova et. al | Yes | Yes | Yes | Yes | No | Yes | Yes | Yes | Yes |
| Ji et. al | Yes | No | Yes | Yes | Yes | Yes | Yes | Yes | Yes |
| Jit et. al | Yes | Yes | Yes | Yes | No | Yes | Yes | Yes | Yes |
| Jo et. al | Yes | No | Yes | Yes | Yes | Yes | Yes | Yes | Yes |
| Karki et. al | No | Yes | Yes | Yes | No | Yes | No | Yes | Yes |
| Kranzer et. al | Yes | No | Yes | Yes | Yes | Yes | Yes | Yes | Yes |
| Machekera et. al | Yes | Yes | Yes | Yes | No | Yes | Yes | Yes | Yes |
| Maheswaran et. al | Yes | Yes | Yes | Yes | No | Yes | Yes | Yes | Yes |
| Murray et. al | Yes | Yes | Yes | Yes | Yes | Yes | Yes | Yes | Yes |
| Orlando et. al | Yes | Yes | Yes | Yes | No | Yes | Yes | Yes | Yes |
| Reddy et. al | Yes | Yes | Yes | Yes | Yes | Yes | Yes | Yes | Yes |
| Sekandi et. al | Yes | Yes | Yes | Yes | No | Yes | Yes | Yes | Yes |
| Shah et. al (Peru) | Yes | Yes | Yes | Yes | No | Yes | Yes | Yes | Yes |
| Shah et. al (Ethiopia) | Yes | Yes | Yes | Yes | Yes | Yes | Yes | Yes | Yes |
| Shah et. al (Vietnam) | Yes | No | Yes | Yes | No | Yes | Yes | Yes | Yes |
| Smit et. al | Yes | No | Yes | Yes | Yes | Yes | Yes | Yes | Yes |
| Sohn et. al | Yes | No | Yes | Yes | No | Yes | Yes | Yes | Yes |
| Winetsky et. al | Yes | Yes | Yes | Yes | Yes | Yes | Yes | Yes | Yes |
| Yoon et. al | Yes | Yes | Yes | Yes | No | Yes | Yes | Yes | Yes |
| Zhang et. al | Yes | No | Yes | Yes | No | Yes | Yes | Yes | Yes |
| Zishiri et. al | Yes | No | Yes | Yes | No | Yes | Yes | Yes | Yes |
| Zwerling et. al | Yes | Yes | Yes | Yes | Yes | Yes | Yes | Yes | Yes |

Supplemental Table 2: Quality Assessment Part II

| **Study Author** | **Are all important and relevant outcomes for each alternative identified?** | **Are all outcomes measured appropriately in physical units?** | **Are outcomes valued appropriately?** | **Is an incremental analysis of costs and outcomes of alternatives performed?** | **Are all future costs and outcomes discounted appropriately?** | **Are all important variables, whose values are uncertain, appropriately subjected to sensitivity analysis?** | **Do the conclusions follow from the data reported?** | **Does the study discuss the generalizability of the results to other settings and patient/client groups?** | **Does the article indicate that there is no potential conflict of interest of study researcher(s) and funder(s)?** |
| --- | --- | --- | --- | --- | --- | --- | --- | --- | --- |
| Abimbola et. al | Yes | Yes | Yes | Yes | No | Yes | Yes | Yes | Yes |
| Adelman et. al | Yes | Yes | Yes | Yes | No | Yes | Yes | Yes | Yes |
| Andrews et. al | Yes | Yes | Yes | Yes | Yes | Yes | Yes | Yes | Yes |
| Bassett et. al | No | No | No | Yes | No | No | Yes | Yes | Yes |
| Bogdanova et. al | Yes | Yes | No | Yes | No | No | Yes | No | Yes |
| Ji et. al | Yes | Yes | Yes | Yes | No | Yes | Yes | No | Yes |
| Jit et. al | Yes | Yes | Yes | Yes | Yes | Yes | Yes | Yes | Yes |
| Jo et. al | Yes | Yes | No | Yes | Yes | No | Yes | Yes | Yes |
| Karki et. al | Yes | Yes | No | Yes | No | No | Yes | Yes | Yes |
| Kranzer et. al | Yes | Yes | No | Yes | Yes | Yes | Yes | Yes | Yes |
| Machekera et. al | Yes | Yes | No | Yes | No | Yes | Yes | Yes | Yes |
| Maheswaran et. al | Yes | Yes | Yes | Yes | Yes | Yes | Yes | Yes | Yes |
| Murray et. al | Yes | Yes | Yes | Yes | Yes | Yes | Yes | Yes | Yes |
| Orlando et. al | Yes | Yes | Yes | Yes | Yes | Yes | Yes | Yes | No |
| Reddy et. al | Yes | Yes | Yes | Yes | Yes | Yes | Yes | Yes | Yes |
| Sekandi et. al | Yes | Yes | No | Yes | No | Yes | Yes | Yes | Yes |
| Shah et. al (Peru) | Yes | Yes | Yes | Yes | No | Yes | Yes | Yes | Yes |
| Shah et. al (Ethiopia) | Yes | Yes | No | Yes | No | No | Yes | No | Yes |
| Shah et. al (Vietnam) | Yes | Yes | No | Yes | No | No | Yes | Yes | Yes |
| Smit et. al | Yes | Yes | No | Yes | No | No | Yes | Yes | Yes |
| **Study Author** | **Are all important and relevant outcomes for each alternative identified?** | **Are all outcomes measured appropriately in physical units?** | **Are outcomes valued appropriately?** | **Is an incremental analysis of costs and outcomes of alternatives performed?** | **Are all future costs and outcomes discounted appropriately?** | **Are all important variables, whose values are uncertain, appropriately subjected to sensitivity analysis?** | **Do the conclusions follow from the data reported?** | **Does the study discuss the generalizability of the results to other settings and patient/client groups?** | **Does the article indicate that there is no potential conflict of interest of study researcher(s) and funder(s)?** |
| Sohn et. al | Yes | Yes | No | Yes | No | Yes | Yes | Yes | No |
| Winetsky et. al | Yes | Yes | Yes | Yes | Yes | Yes | Yes | Yes | Yes |
| Yoon et. al | Yes | Yes | No | Yes | No | No | Yes | Yes | Yes |
| Zhang et. al | Yes | Yes | No | Yes | No | No | Yes | Yes | Yes |
| Zishiri et. al | Yes | Yes | No | Yes | No | Yes | Yes | Yes | Yes |
| Zwerling et. al | Yes | Yes | Yes | Yes | Yes | Yes | Yes | Yes | Yes |


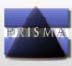
**PRISMA 2009 Checklist**

| **Section/topic** | **#** | **Checklist item** | **Reported on page #** |
| --- | --- | --- | --- |
| **TITLE** | | |  |
| Title | 1 | Identify the report as a systematic review, meta-analysis, or both. | 1 |
| **ABSTRACT** | | |  |
| Structured summary | 2 | Provide a structured summary including, as applicable: background; objectives; data sources; study eligibility criteria, participants, and interventions; study appraisal and synthesis methods; results; limitations; conclusions and implications of key findings; systematic review registration number. | 2-3 |
| **INTRODUCTION** | | |  |
| Rationale | 3 | Describe the rationale for the review in the context of what is already known. | 4-5 |
| Objectives | 4 | Provide an explicit statement of questions being addressed with reference to participants, interventions, comparisons, outcomes, and study design (PICOS). | 5, Supplement |
| **METHODS** | | |  |
| Protocol and registration | 5 | Indicate if a review protocol exists, if and where it can be accessed (e.g., Web address), and, if available, provide registration information including registration number. | 5 |
| Eligibility criteria | 6 | Specify study characteristics (e.g., PICOS, length of follow-up) and report characteristics (e.g., years considered, language, publication status) used as criteria for eligibility, giving rationale. | 5-6 |
| Information sources | 7 | Describe all information sources (e.g., databases with dates of coverage, contact with study authors to identify additional studies) in the search and date last searched. | 5-6 |
| Search | 8 | Present full electronic search strategy for at least one database, including any limits used, such that it could be repeated. | 5-6, Supplement |
| Study selection | 9 | State the process for selecting studies (i.e., screening, eligibility, included in systematic review, and, if applicable, included in the meta-analysis). | 6 |
| Data collection process | 10 | Describe method of data extraction from reports (e.g., piloted forms, independently, in duplicate) and any processes for obtaining and confirming data from investigators. | 6 |
| Data items | 11 | List and define all variables for which data were sought (e.g., PICOS, funding sources) and any assumptions and simplifications made. | 7 |
| Risk of bias in individual studies | 12 | Describe methods used for assessing risk of bias of individual studies (including specification of whether this was done at the study or outcome level), and how this information is to be used in any data synthesis. | N/A |
| Summary measures | 13 | State the principal summary measures (e.g., risk ratio, difference in means). | 7 |
| Synthesis of results | 14 | Describe the methods of handling data and combining results of studies, if done, including measures of consistency (e.g., I^2^) for each meta-analysis. | 7 |
